# MRI to guide clinical management of rectal cancer: updated consensus recommendations from the European Society of Gastrointestinal and Abdominal Radiology (ESGAR): PART II—Restaging and response evaluation

**DOI:** 10.1007/s00330-025-12275-9

**Published:** 2026-01-29

**Authors:** ESGAR Rectal Imaging Guideline Group, Juan-Ramón Ayuso, Svetlana Balyaniskova, Regina G. H. Beets-Tan, Ivana Blazic, Lennart Blomqvist, Damiano Caruso, Filippo Crimì, Luís Curvo-Semedo, Raphaëla C. Dresen, Marc J. Gollub, Vicky Goh, Kirsten Gormly, Sofia Gourtsoyianni, Bengi Gurses, Christine Hoeffel, Andreas M. Hötker, Natally Horvat, Davide Ippolito, Seung Ho Kim, Andrea Laghi, Max J. Lahaye, Doenja M. J. Lambregts, Monique Maas, Stephanie Nougaret, Cinthia D. Ortega, Emilio Quaia, Søren R. Rafaelsen, Pablo Rodríguez Carnero, Inês Santiago, Saugata Sen, Soleen Stocker-Ghafoor, Jaap Stoker

**Affiliations:** 1 Hospital Clinic, Barcelona, Spain; 2 Kingston and Richmond NHS Foundation Trust, London, UK; 3 The Netherlands Cancer Institute, Amsterdam, The Netherlands; 4 Clinical Hospital Centre Zemun, Belgrade, Serbia; 5 Karolinska Institutet, Stockholm, Sweden; 6 Department of Medical Surgical Sciences and Translational Medicine, Sapienza University of Rome, Rome, Italy; 7 University of Padova, Padova, Italy; 8 Department of Imaging, Local Health Unit-Aveiro Region and Faculty of Medicine, University of Coimbra, Coimbra, Portugal; 9 University Hospitals Leuven, Leuven, Belgium; 10 Memorial Sloan Kettering Cancer Center, New York, USA; 11 School of Biomedical Engineering & Imaging Sciences, King’s College London, London, UK; 12 The University of Adelaide, Adelaide, Australia; 13 1st Department of Radiology NKUA, Areteion Hospital, Athens, Greece; 14 Department of Radiology, Koc University School of Medicine, Istanbul, Turkey; 15 Université de Reims, Champagne-Ardennes, CHU Reims, CRESTIC, Reims, France; 16 University Hospital Zürich, Zürich, Switzerland; 17 Mayo Clinic, Rochester, USA; 18 School of Medicine and Surgery, University of Milan-Bicocca, Monza, Italy; 19 Inje University Haeundae Paik Hospital, Busan, Korea; 20 Humanitas University, Department of Biomedical Sciences, and IRCCS Humanitas Research Hospital, Radiology Department, Milan, Italy; 21 Montpellier Cancer Center, PINKCC Lab, U1194 Montpellier, France; 22 Hospital das Clinicas HCFMUSP, Faculdade de Medicina, Universidade de Sao Paulo, Hospital Israelita Albert Einstein, Sao Paulo, Brazil; 23 University Hospital of Southern Denmark, Vejle, Denmark; 24 La Princesa University Hospital, Madrid, Spain; 25 Hospital da Luz, Lisbon, Portugal; 26 Tata Medical Center, Kolkata, India; 27 Amsterdam University Medical Centre, Amsterdam, The Netherlands

**Keywords:** Rectal cancer, Magnetic resonance imaging, Clinical guidelines, Restaging

## Abstract

**Objectives:**

To provide up-to-date consensus recommendations on the acquisition, interpretation and reporting of MRI for restaging and response evaluation of rectal cancer after neoadjuvant treatment.

**Materials and methods:**

A panel of twenty-six abdominal imaging experts from the European Society of Gastrointestinal and Abdominal Radiology (ESGAR) participated in an online consensus process, led by three independent non-voting chairs. The process adhered to an adapted version of the RAND-UCLA Appropriateness Method. A total of 126 items were scored (22 general, 55 on primary staging, and 49 on restaging after neoadjuvant treatment), and classified using a cut-off of ≥ 80% to establish consensus.

**Results:**

Consensus was reached for 121 items (96%), from which recommendations regarding hardware, patient preparation, image acquisition protocols, criteria for image interpretation, and MRI reporting were constructed. The current manuscript addresses the results related to restaging after neoadjuvant treatment. Only 1/49 restaging items did not reach consensus. Compared to the previous guideline editions, updated and more detailed recommendations were established on how to assess fibrosis after neoadjuvant therapy, how to restage in the setting of organ preservation, the use of tumour regression grading systems, response assessment in mucinous tumours, evaluation of mesorectal fascia (MRF) involvement and presence of extramural venous invasion (EMVI) after neoadjuvant treatment, and how to deal with nodal response for defining the ycN-category after treatment.

**Conclusions:**

These updated expert consensus recommendations serve as clinical guidelines for the restaging of rectal cancer after neoadjuvant treatment using MRI. Recommendations for primary staging are addressed in a separate publication.

**Key Points:**

***Question****Since the last ESGAR rectal imaging guideline update, the rectal cancer treatment landscape has further evolved, necessitating updates to the existing guidelines*.

***Findings****An online consensus process involving 26 panellists led to 96% consensus across 121 items discussed, including 49 items related to restaging after neoadjuvant treatment*.

***Clinical relevance****Key updates included in these updated guidelines for MRI restaging of rectal cancer include new recommendations for assessing fibrosis, identifying patients for organ preservation, use of tumour regression grading systems, assessing mucinous tumours, ycEMVI, ycMRF, and ycN assessment*.

**Graphical Abstract:**

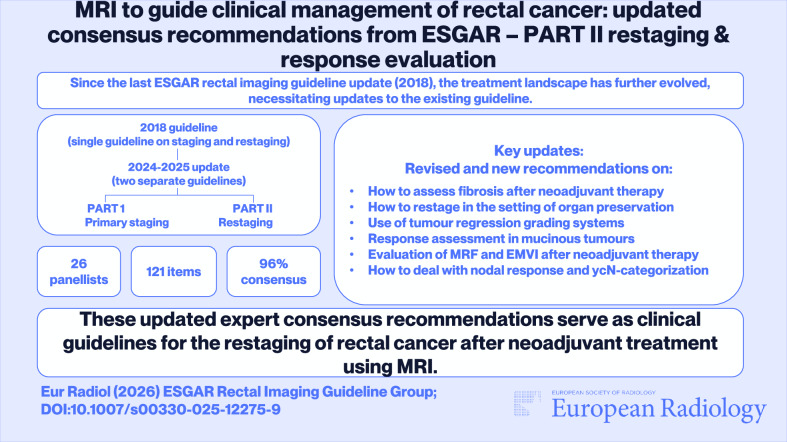

## Introduction

In 2013, the European Society of Gastrointestinal and Abdominal Radiology (ESGAR) published its first consensus recommendations on MRI for staging rectal cancer [1]. An updated version was published in 2018, introducing structured template reports for primary (baseline) staging and restaging after neoadjuvant treatment, including detailed recommendations on lymph node staging and the use of diffusion-weighted imaging [2]. The guideline has now undergone another update, which is divided into two separate publications. The first part contains recommendations for MRI acquisition, interpretation and reporting during primary staging and is presented in a separate publication [3]. The current paper (part II) addresses recommendations for restaging and response evaluation after neoadjuvant treatment. This guideline does not comprise recommendations on multidisciplinary management and follow-up during organ preservation.

## Methods

A modified version of the RAND-UCLA Appropriateness Method was chosen as the consensus-building method for this update. The detailed steps of the guideline process are outlined in the companion guideline publication (part I) on primary staging [3], and can be summarised as follows:

- Step 1—Panel selection: 26 panellists (J.-R.A., S.B., I.B., L.B., D.C., L.C.-S., R.D., M.G., V.G., K.G., S.G., B.G., C.H., A.H., N.H., D.I., S.K., A.L., M.L., S.N., C.O., E.Q., S.R., I.S., S.S., J.S.) who composed the voting panel were selected by the steering group, led by three non-voting guideline chairs (D.L., M.M., R.B.-T.). Three junior fellows (F.C., P.R.C., S.S.-G.) assisted with the literature review and evidence synthesis.
- Step 2—First online questionnaire and voting round: recommendations from the 2018 guidelines were adopted or discarded by the panel, and additional questionnaire topics were identified.
- Step 3—Literature review and evidence synthesis: resulting in a final evidence synthesis document with draft statements and corresponding levels of evidence (see Supplement 1).
- Step 4—Second online questionnaire and voting round: panellists voted on the draft statements (agree/disagree/uncertain) and provided suggestions for improvement. Due to the low level of disagreement, an additional voting round or face-to-face meeting was deemed unnecessary.
- Step 5—Data analysis and reporting: final recommendations (including level of consensus) were established and refined based on panel input.

## Results

Detailed demographic data on the panellists and their local practice, as well as a guideline process flowchart, are provided in the companion publication on primary staging [3]. The panel included 13 male and 13 female radiologists from 18 different countries.

### Areas of consensus

**Fig. 1:**
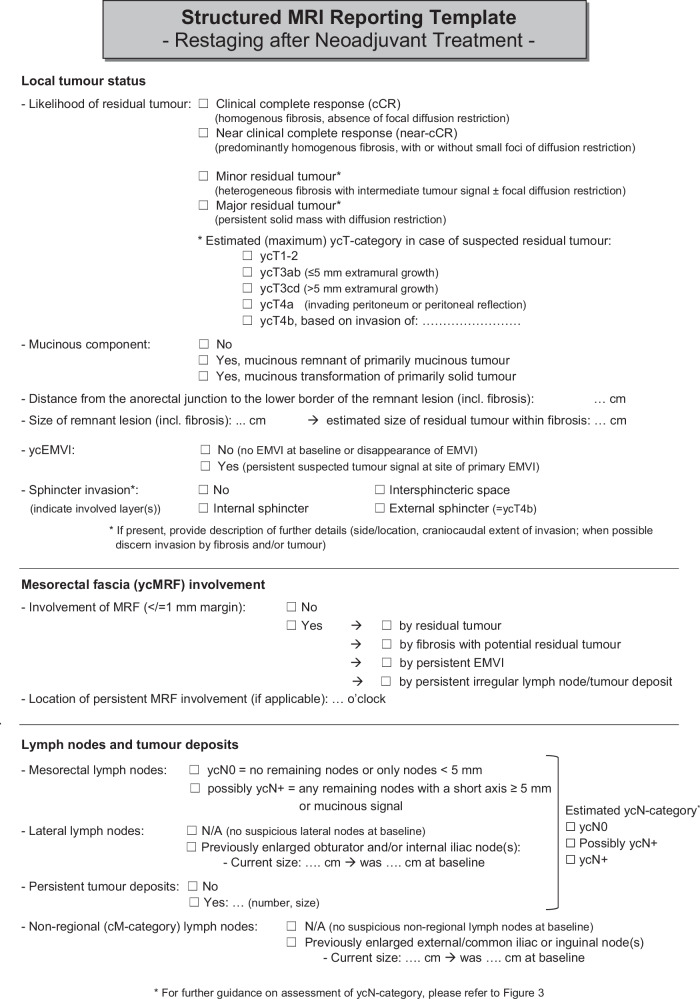
Recommended structured reporting template for restaging after neoadjuvant treatment. EMVI, extramural vascular invasion; yc-suffix, represents restaging of a feature after neoadjuvant treatment based on clinical and/or imaging assessment

In summary, the guideline process resulted in 121 statements (96%) for which consensus was reached and five non-consensus items (4%). The following sections elaborate on the outcomes of the 49 items related to restaging after neoadjuvant treatment. Panellists reached ≥ 80% consensus on 48 of the 49 items (98%). A summary of the recommendations and updates compared to the 2018 edition is provided in Table 1. The proposed updated restaging template for structured reporting, endorsed by the panel, is provided in Fig. 1.

**Table 1:** Synopsis and key recommendations for MRI restaging of rectal cancer (based on items for which ≥ 80% consensus was reached)

I—Recommendations for MR image acquisition	% consensus
*Hardware, protocol and patient preparation*	
- MRI should routinely be performed for restaging of rectal cancer after neoadjuvant treatment	100%
- The standard MRI protocol for restaging should routinely include a DWI sequence	96%
- **A preparatory micro-enema is recommended for restaging to reduce susceptibility artefacts on DWI**	**85%**
- **DWI is mandatory for tumour response assessment as it significantly enhances the performance of MRI to discern between residual tumour and complete response after neoadjuvant treatment**	**89%**
- **For tumour response assessment, reduced FOV DWI is recommended; full FOV acquisitions are optional to ensure complete assessment of the whole pelvis, including all pelvic nodal stations**	**92%**
- **For tumour response assessment, DWI should be acquired in the same plane as the axial T2-weighted sequence (oblique-axial, perpendicular to the tumour axis)**	**81%**
- Further protocol recommendations are the same as for primary staging (field strength 1.5–3.0 T using an external surface coil; 2D T2W sequences of the tumour in 3 planes with slice thickness 3 mm and in-plane resolution < 1 × 1 mm; large FOV T2W or T1W sequence covering all pelvic compartments), as detailed in the publication on primary staging (3)	92–100%

II—Recommendations for MR image interpretation and reporting	
*General (overall response assessment)*	
- Structured reporting of rectal cancer MRI is recommended and should include the items described in the structured reporting template for restaging presented in Fig. 1.	96%
- On restaging MRI, a normalised two-layered appearance of the rectal wall on T2W MRI is suggestive of a complete response	96%
- On restaging MRI, a homogeneous hypointense fibrotic residue on T2W MRI without any isointense mass or signal is indicative of a near-complete or complete response	88%
- **T2-weighted MRI can accurately select patients with major residual tumour who will require radical surgery (total mesorectal excision)**	**100%**
- **The findings of T2-weighted MRI should be combined with those of DWI and endoscopy to assess a (near) complete response after neoadjuvant treatment**	**92%**
- **DWI should be assessed in conjunction with T2-weighted MRI**	**100%**
- **Response evaluation after neoadjuvant CRT should include an estimation of the degree and pattern of fibrosis, combined with the presence and pattern of diffusion restriction**	**92%**
- **The same criteria to assess response after neoadjuvant (chemo)radiotherapy are also recommended for patients undergoing alternative neoadjuvant treatments (e.g., TNT)**	**89%**
- **Mucinous degeneration in a primarily non-mucinous tumour should not be regarded as a sign of non-response**	**100%**
- **A restaging MRI report should include an overall classification of response to help inform further treatment planning and should be classified as (near-)complete response, minor residual tumour, or major residual tumour**	**89%**
- **mrTRG is useful to estimate the overall degree of response (good versus poor), but is not accurate to identify patients with a complete response**	**89%**
*Tumour location and ycT-category*	
- **Tumour height and length measurements after neoadjuvant treatment should encompass the fibrotic remnants of the tumour bed**	92%
- **In case of a suspected (near)complete response after neoadjuvant treatment, detailed reporting of the ycT-category is not recommended as it is unreliable and has no clinical implications.**	81%
- **In patients with suspected residual tumour after neoadjuvant treatment, the ycT-category should be reported as an estimation (representing the maximum ycT-category) and encompass any fibrotically changed areas of the former tumour bed**	85%
- **DWI is not recommended to determine the ycT-category, except to assess ycT0 versus ycT**+	96%
*Mesorectal fascia (ycMRF) and extramural vascular invasion (ycEMVI)*	
- If a fatpad reappears between the tumour and MRF after neoadjuvant treatment, the MRF should be reported as cleared / uninvolved (ycMRF-)	100%
- Persistent stranding into the MRF after neoadjuvant treatment should be considered an equivocal sign that may or may not indicate persistent MRF involvement	96%
- **The same grading system should be applied for both primary EMVI assessment and EMVI assessment following neoadjuvant treatment**	89%
- **DWI may be of added value to assess ycEMVI and ycMRF status after CRT**	85%
*Lymph nodes (ycN-category)*	
- **A size cut-off of 5 mm (short axis) may be used to restage mesorectal lymph nodes following neoadjuvant treatment, while being mindful of its limitations**	92%
- **When considering patients for organ preservation, MRI may be used to monitor nodal growth (‘test of time’) and identify ycN+ disease**	96%
- **There are currently no recommended size thresholds or other criteria for evaluation of lateral lymph nodes after neoadjuvant treatment**	85%

Recommendations in bold font represent updates or additions to recommendations made in the previous guideline edition

Only one statement did not reach consensus (73%): ‘It is recommended to use the same response criteria after neoadjuvant treatment for both solid tumours and mucinous tumours’

*CRT* chemoradiation/chemoradiotherapy, *DWI* diffusion–weighted imaging, *FOV* field–of–view, *TNT* total neoadjuvant therapy, *yc–suffix* represents restaging of a feature after neoadjuvant treatment based on clinical and/or imaging assessment

### Areas lacking consensus

Only one restaging statement lacked consensus, though moderate agreement (73%) was reached. This concerned the statement ‘It is recommended to use the same response criteria after neoadjuvant treatment for both solid and mucinous tumours’.

### Main changes and additions to the previous guideline edition

**Fig. 2:**
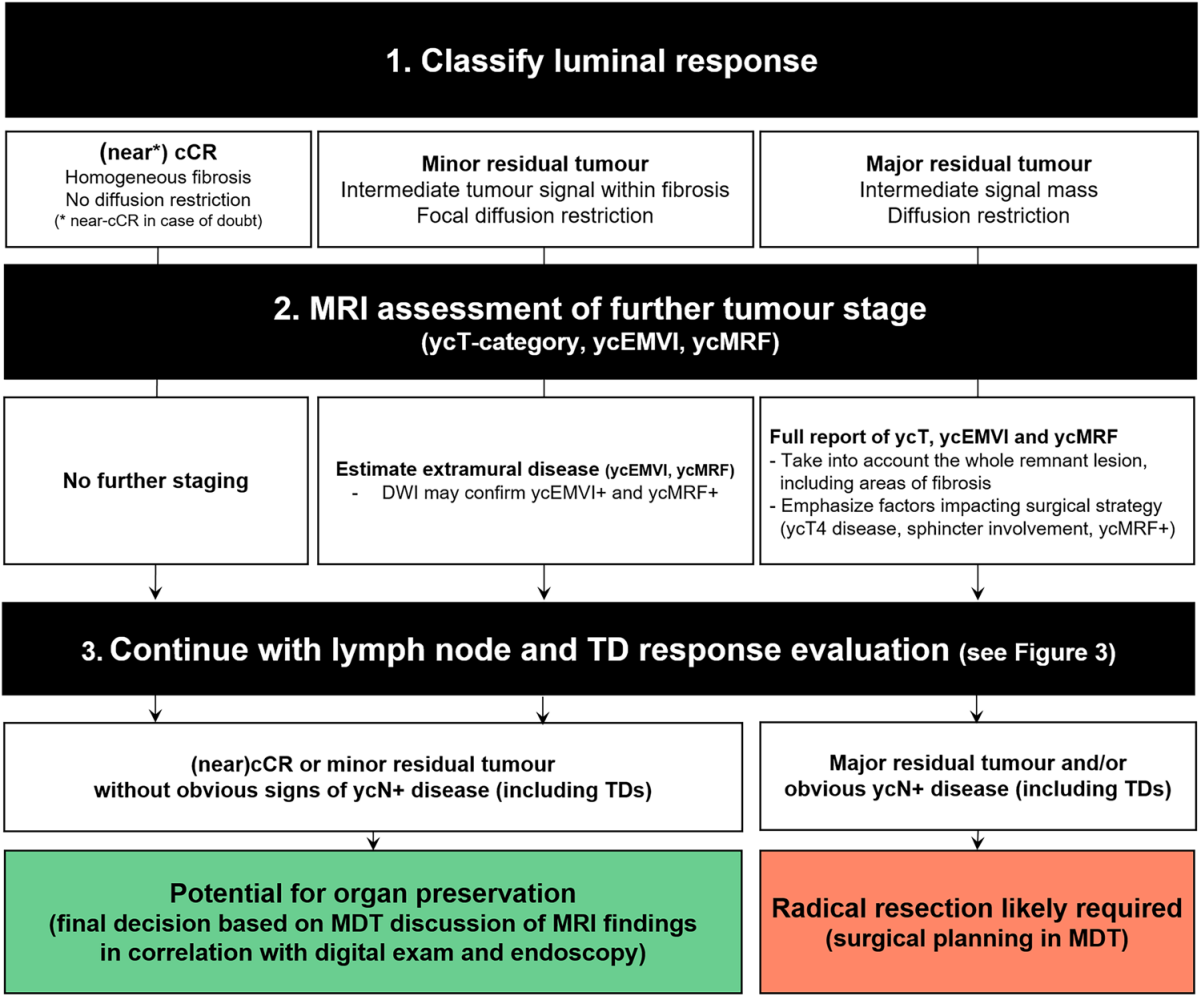
Stepwise approach to adaptive restaging of rectal cancer tailored to the current treatment landscape (including organ preservation). cCR, clinical complete response; EMVI, extramural vascular invasion; MDT, multidisciplinary team; MRF, mesorectal fascia; TD, tumour deposit; yc–suffix, represents restaging of a feature after neoadjuvant treatment based on clinical and/or imaging assessment

**Fig. 3:**
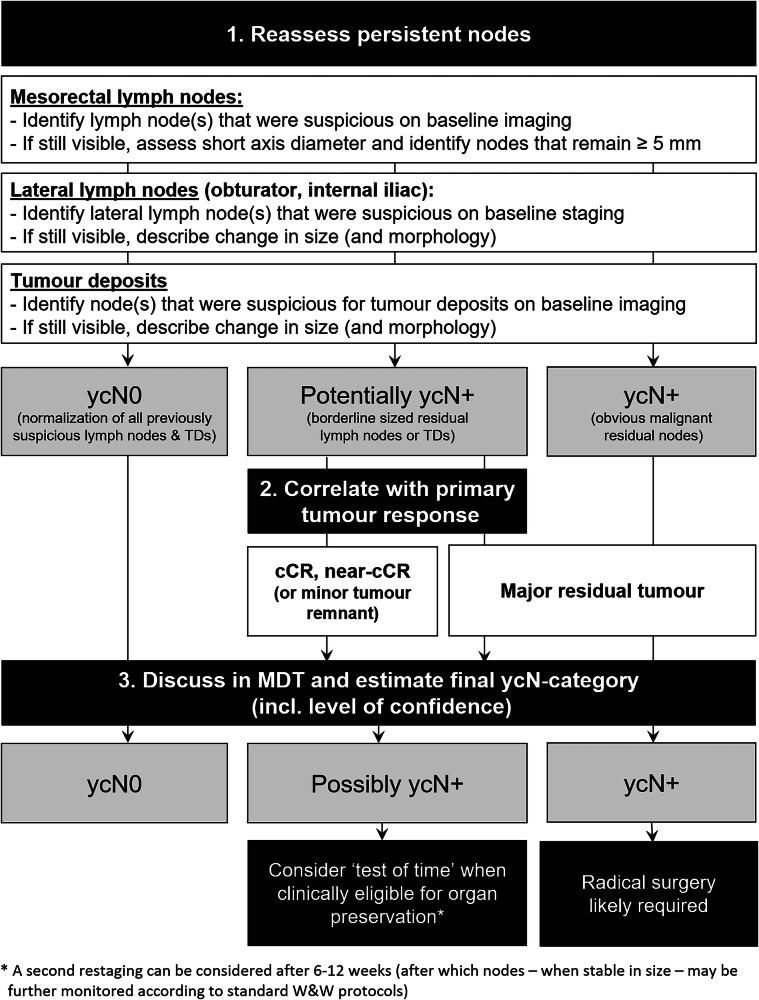
Stepwise approach for response assessment of mesorectal lymph nodes, lateral lymph nodes and tumour deposits (ycN–category). cCR, clinical complete response; MDT, multidisciplinary team; TD, tumour deposit; W & W, watch–and–wait; yc–suffix, represents restaging of a feature after neoadjuvant treatment based on clinical and/or imaging assessment

The main changes and additions to the 2018 guidelines include more detailed recommendations on how to assess fibrosis after neoadjuvant therapy, how to restage in the setting of organ preservation, the use of tumour regression grading systems, response assessment in mucinous tumours, how to assess ycMRF involvement and ycEMVI, and how to deal with nodal response and ycN-categorisation. Table 2 provides an overview of the key updates and recommendations, accompanied by explanatory notes detailing their rationale. A stepwise flowchart with recommendations on how to perform adaptive restaging within the clinical context of organ preservation is provided in Fig. 2. Figure 3 provides a practical, stepwise approach for nodal response assessment and estimation of the ycN-category to guide clinical management after neoadjuvant treatment.

**Table 2:** Summary of key recommendations, changes and updates to the previous guideline edition specific to restaging after neoadjuvant treatment

Topic	Updated recommendation*	Old recommendation (from 2016 consensus meeting)	Explanatory notes
Patient preparation	• A preparatory micro-enema is recommended for restaging to reduce susceptibility artefacts on DWI	• Use of an enema is not routinely recommended	• The use of an enema in a restaging setting has been shown to improve image quality of the DWI sequence [4, 5]
MR protocol for restaging	• In-plane resolution for T2W MRI should be < 1 × 1 mm	• N/A (New recommendation)	
	• Protocol should include a large FOV T2W or T1W sequence covering all relevant pelvic lymph node compartments	• N/A (New recommendation)	
	• Reduced FOV DWI is recommended; full FOV acquisitions are optional to include all pelvic nodal stations	• N/A (New recommendation)	
	• DWI should be angled in the same oblique-axial plane as T2W MRI	• N/A (New recommendation)	
	• DWI is mandatory to increase accuracy to discern between residual tumour and complete response	• N/A (New recommendation)	• A pattern-based approach that integrates T2W imaging and DWI has been adopted as the recommended approach for response assessment in the reporting template (Fig. 1)
MR response assessment	• DWI should be assessed in conjunction with T2W MRI; findings of T2W MRI should be combined with those of DWI and endoscopy to assess a (near) complete response	• N/A (New recommendation)	• The combination of digital rectal exam, MRI and endoscopy yields the best results to identify patients with a complete response and is therefore recommended when considering organ preservation
	• Response evaluation after neoadjuvant CRT should include an estimation of the degree and pattern of fibrosis, combined with the presence and pattern of diffusion restriction	• N/A (New recommendation)	
	• The same criteria to assess response after neoadjuvant (chemo)radiotherapy are also recommended for patients undergoing alternative neoadjuvant treatments (e.g., TNT)	• N/A (New recommendation)	• No evidence exists to support a different approach
	• It is recommended to use the same response criteria after neoadjuvant treatment for both solid tumours and mucinous tumours (73% consensus)	• N/A (New recommendation)	• The panel acknowledges that these criteria are suboptimal in mucinous (or mixed) tumours, but adopted them with moderate consensus, given the lack of validated alternative response criteria for mucinous tumours. It should be considered that the overall risk of residual tumour is higher in mucinous tumours than in solid tumours.
	• A restaging MRI report should include an overall classification of response into the following categories: (near-)CR, minor residual tumour or major residual tumour	• N/A (New recommendation)	
	• mrTRG is useful to estimate the overall degree of response (good vs. poor) but cannot accurately identify a CR	• N/A (New recommendation)	• mrTRG is not recommended as part of the structured reporting template, which recommends an integrated assessment of T2W and DWI (the latter is not included in mrTRG) to stratify patients according to their response
	• Tumour height and length measurements after neoadjuvant treatment should encompass the fibrotic remnants of the tumour bed	• N/A (New recommendation)	• In case of residual tumour within fibrosis, the residual tumour areas are often scattered. As such, the whole fibrotic remnant will be resected during TME.
ycT-category	• In case of a suspected (near-)CR, detailed ycT-category reporting is not recommended as it is unreliable and has no clinical implications	• A restaging report should include yT-stage classified as ycT0, ycT1-2, ycT3 and ycT4	
	• In patients with suspected residual tumour, the ycT-category should be reported as an estimation (representing the maximum ycT) and encompass any fibrotically changed areas of the former tumour bed	• N/A (New recommendation)	
	• DWI is not recommended for ycT-categorisation, except to assess ycT0 versus ycT+	• DWI is not accurate in discriminating between ycT1-2 and ycT3-4 tumours • DWI is accurate in discriminating between CR and residual tumour	
ycMRF & ycEVMI	• The same grading system should be applied for both primary EMVI staging and ycEMVI restaging following neoadjuvant treatment	• N/A (New recommendation)	• Grade 3–4 ycEMVI should be regarded as ycEMVI+. However, ycEMVI assessment is more challenging in the case of fibrosis.
	• DWI may be of added value to assess ycEMVI and ycMRF	• DWI is not accurate to assess ycEMVI and ycMRF	
Lymph nodes & tumour deposits (ycN-category)	• A size cut-off of 5 mm (short axis) may be used to restage mesorectal lymph nodes following neoadjuvant treatment, while being mindful of its limitations	• All nodes with a short-axis diameter < 5 mm should be considered benign • For nodes with a short-axis diameter ≥ 5 mm, no reliable criteria exist	• As outlined in Fig. 2, the panel recommends estimating the ycN-category in correlation with the primary tumour response, as nodal response is often greater or at least similar to luminal response.
	• When considering patients for organ preservation, MRI may be used to monitor nodal growth (‘test of time’) and identify ycN+ disease	• N/A (New recommendation)	• No strict evidence-based protocol for follow-up in doubtful nodes is available. A second nodal restaging can be considered after 6–12 weeks. If nodes remain stable or show further response, the MDT may consider a watch-and-wait approach
	• There are currently no recommended size thresholds or other criteria for lateral nodal restaging after neoadjuvant treatment	• N/A (New recommendation)	• It is advised to describe the response (change in size) and consider it in correlation with the response of the primary tumour and other (mesorectal) lymph nodes and TDs. In case of an overall good response, a ‘test of time’ could be considered to monitor the lateral nodes, after discussion in the MDT.

* Unless otherwise indicated, recommendations presented in this table achieved ≥ 80% consensus

*CR* complete response, *CRT* chemoradiation/chemoradiotherapy, *DWI* diffusion-weighted imaging, *EMVI* extramural vascular invasion, *FOV* field of view, *MRF* mesorectal fascia, *mrTRG MRI* tumour regression grade, *TME* total mesorectal excision, *TNT* total neoadjuvant therapy, *yc–suffix* represents restaging of a feature after neoadjuvant treatment based on clinical and/or imaging assessment

## Discussion

### Imaging techniques and patient preparation

The recommended restaging MRI protocol largely mirrors that of primary staging. Specific protocol recommendations can be found in the companion guideline document on primary staging (Part I) [3]. One restaging-specific recommendation that was agreed upon by 85% of the panellists is to use a micro-enema before acquiring the restaging MRI to reduce susceptibility artefacts on DWI [4, 5].

### Adaptive strategy for restaging in the setting of organ preservation: a response-based classification

Since the previous guidelines were published in 2018, significant progress has been made in assessing rectal cancer response following neoadjuvant therapy. A key driver of this advancement has been the increased worldwide adoption of organ-preserving treatment strategies, which require a revised approach to restaging [6, 7]. One of the main responsibilities of a radiologist in a multidisciplinary management team is to evaluate response on MRI to identify patients who may be candidates for organ preservation—i.e., watch-and-wait (W&W) or additional local therapy—and differentiate these patients from poorly or partially responding patients who require radical resection. Although terminology used to classify response groups varies widely in published literature and no uniform criteria exist to define a near-complete response versus minor residual tumour [8–10], the panel agreed that it would make sense to adopt a response classification system that is closely linked to treatment implications, as outlined in Fig. 2. The panel recommends that radiologists classify patients into three main response categories: (1) (clinical) complete or near-complete response (CR/near-CR), encompassing all patients that could potentially be considered for watch-and-wait, (2) minor residual tumour (i.e., patients potentially eligible for local treatment) and (3) major residual tumour (i.e., patients requiring radical resection). MRI criteria to categorise patients into these response groups are explained in the reporting template (Fig. 1). It is important to clearly identify patients who are potentially eligible for organ preservation in the conclusion of the MRI report.

The panel further reiterates previous guideline recommendations that response assessment should be based on an integrated assessment of T2-weighted MRI, DWI and endoscopy. When a complete or near-complete response is observed, radiologists should refrain from assigning a ycT-category. This recommendation differs from the 2018 guideline edition, which still recommended ycT-categorisation in all cases. Assigning a ycT-category in (near-) complete responders leads to low accuracy, has limited clinical implications and can be confusing, especially when categories are expressed as a wide range (e.g., ycT0-3ab) [11]. This could potentially lead to an incorrect disqualification of patients for organ preservation. Other common interpretation pitfalls include false positive findings on DWI caused by “T2-shine through”, susceptibility artefacts, or non-tumoural signal in areas of inflammation or ulceration. A critical evaluation of DWI findings in conjunction with the ADC map, taking into account the shape of the DWI signal, can be helpful to prevent interpretation errors [12]. During response evaluation, radiologists should be aware that the apparent tumour response on MRI and endoscopy often lags behind the true histological response, meaning that residual abnormalities on MRI may persist even when there is minimal or no viable tumour tissue remaining. In case of doubt or clinical near-complete response, a second restaging with endoscopy and MRI can be performed (after an extended observation period of another 6–12 weeks) to enable a final diagnosis of CR or residual tumour [13]. In patients with major residual tumour, restaging remains essential for surgical planning in cases with significant residual tumour, with emphasis on the assessment of the surgical resection margins.

### Tumour response grading systems

Several response grading systems exist, including the MRI tumour regression grade (mrTRG) that classifies the degree of residual tumour versus fibrosis on T2W-MRI, and modified versions of the mrTRG that combine the mrTRG with the presence of high signal (indicative of tumour) on DWI [14–16]. The panel agreed that the use of DWI is mandatory in response evaluation and that patterns such as residual suspicious (intermediate) signal on T2W MRI, fibrosis and diffusion restriction should be combined to assess response, regardless of the type of neoadjuvant treatment used. This combined assessment of T2W MRI and DWI was incorporated in the clinical response classification system described above and included in the reporting template in Fig. 1. The consensus was that mrTRG can provide a general estimate of the response (poor versus good). However, similar to other response grading systems, mrTRG lacks adequate sensitivity to reliably identify pathological complete responders [16–19]. As such, the panel chose not to include mrTRG as a separate parameter in the reporting template.

### Assessing mucinous tumours after neoadjuvant treatment

The current guideline update specifically addresses mucinous tumours and mucinous degeneration. No consensus was reached on how to evaluate the response of mucinous tumours. Most panellists (73%) recommended applying the same response criteria as those used for solid tumours. However, based on individual comments received from panellists, this recommendation appears to come from the lack of dedicated response criteria for mucinous tumours. The panellists emphasised the clinical need for such dedicated mucinous response criteria, as mucinous tumours behave differently from solid tumours. Mucinous tumours tend to show a limited response to (chemo)radiation, develop minimal fibrosis and show limited utility of DWI [20]. The chance for a complete response is lower than in solid tumours [21]. On MRI (including DWI), it is not possible to distinguish acellular mucin from mucin areas containing viable tumour cells. Park et al developed a tumour regression grading system specifically for mucinous tumours in a small cohort of 59 patients. Although a relationship was found with pathologic TRG, reproducibility was low (Kappa 0.40), and results have not been validated or tested in terms of diagnostic performance [22]. Given these considerations and limitations of MRI, W&W in primary mucinous tumours will be a rare scenario and should always be critically discussed in the MDT.

Mucinous tumours should not be confused with mucinous degeneration (the appearance of acellular mucin) that may occur in some solid tumours after (chemo)radiation. Panellists agreed that this mucinous degeneration—although it should be mentioned in the report—should not be interpreted as a sign of poor response, while acknowledging that its exact clinical relevance remains unclear. A meta-analysis by Reynolds et al found no association between mucinous degeneration and tumour response, recurrence or survival [23]. Clinically, the finding of mucinous degeneration does not immediately preclude watch-and-wait when endoscopy shows a typical complete response [24].

### Approach to restaging residual tumour (height, ycT, ycMRF, and ycEMVI)

The panel agreed that when evaluating the ycT-category and measuring tumour height and length at restaging, the full area of fibrosis should be considered. DWI should only be used to discriminate ycT0 from ycT+ disease and is not suitable to assess the ycT-category in further detail. In contrast to the previous guideline version, this update recognises the potential added value of DWI to assess ycEMVI and ycMRF after neoadjuvant treatment. For ycMRF assessment, the same 1 mm cut-off is advised as for primary staging and a distance of ≤ 1 mm between the MRF and the primary tumour, ycEMVI or any remaining irregular and suspicious tumour deposits or nodes should be regarded as ycMRF+ disease [25]. Morphological assessment of the fibrosis can also offer added benefit. In cases with massive fibrosis extending into the MRF, MRF positivity is frequently encountered at histopathology after surgery, whereas the presence of only minor fibrotic stranding into the MRF after neoadjuvant treatment is usually a sign of a non-involved MRF [26]. Park et al showed that adding DWI improves sensitivity when restaging MRF involvement [27]. For ycEMVI, the same grading system used in primary staging can be applied, where grades 3 and 4 are considered ycEMVI-positive [28]. ycEMVI+ on restaging MRI has been shown to be predictive of worse disease-free survival [28]. Several cohort studies have shown that including DWI can increase the specificity for the assessment of ycEMVI [29–31]. Notably, Kim et al proposed a five-point scoring system incorporating T2W-MRI and DWI to assess viable tumour in EMVI as well as tumour deposits (TDs) following neoadjuvant therapy. This approach demonstrated a sensitivity of 62% and specificity of 93% [31]. In clinical practice, DWI may thus serve as a valuable adjunct to T2W-MRI for the restaging of both ycEMVI and ycMRF status.

### Nodal response assessment (ycN)

In the 2018 guideline, 5 mm was recommended as a practical cut-off to assess mesorectal lymph nodes after CRT, while acknowledging that this may lead to both over- and understaging [2]. In the current guideline update, the 5 mm cut-off was reinstated for mesorectal lymph nodes, and specific considerations have been added regarding nodal response assessment in the context of potential organ preservation (see Fig. 3). When a luminal complete response is found, applying the 5 mm cut-off too strictly may falsely exclude patients from organ preservation. Evidence shows that a luminal response is typically accompanied by a nodal response and that only 7% of patients with a complete luminal response (ypT0) have residual nodal disease (ypN+) [32]. Furthermore, during watch-and-wait, isolated nodal regrowths are very uncommon, constituting only 3–6% of all local regrowths [33, 34]. The likelihood that a “borderline” sized node (of around 5 mm) after neoadjuvant therapy will grow and potentially threaten resection margins is expected to be very low. Therefore, the panel agreed that a cautious approach is advised when basing treatment decisions (i.e., whether or not to opt for organ preservation) on the ycN-category. In patients with a clinical (near)CR of the primary tumour, follow-up with MRI—typically performed after a 6–12 week interval [13, 35]—can be used as a ‘test of time’ to monitor the presence or absence of growth in borderline-sized nodes to ultimately establish a more confident diagnosis of ycN0 versus ycN+ disease. These decisions should always be made by the MDT. The panel did not discuss any specific recommendations for the reassessment of TDs after neoadjuvant treatment, also considering the lack of available scientific literature. Future research will hopefully shed light on how to manage TDs after neoadjuvant treatment. For the time being, TDs will remain part of the ycN-category and changes in the size and appearance of TDs should be reported.

Lateral nodes present a unique challenge for radiologists. While a recommended size cut-off of ≥ 7 mm (short axis) has been established and agreed upon by the panel for primary staging, in the restaging setting, convincing evidence is lacking for a reliable size cut-off or other criteria to assess lateral nodes [36]. Furthermore, there is no consensus on whether to do a lateral lymph node dissection (LLND) after neoadjuvant therapy [37–39]. This leads to a large variation in clinical practice. In practice, there are three potential scenarios after neoadjuvant therapy in patients with lateral node metastases:

1. The lateral node(s) decrease in size (most common scenario)
2. The lateral node(s) disappear
3. The lateral node(s) remain stable in size and morphology, or even progress

Although no specific criteria could be recommended, radiologists should mention the response of the initially suspicious lateral lymph nodes in the MRI report. If the lateral lymph node(s) disappear(s), it would seem logical to assume sterilisation. However, if the lateral node(s) persist(s), the decision on whether LLND is required in individual cases should be made during the MDT discussion. In these discussions, the response of both the primary tumour and mesorectal nodes will be of relevance. Ultimately, decisions will depend on institutional or national protocols, which may vary across regions and hospitals.

### Methodological limitations

Although all panellists completed the second questionnaire and voting round, two panellists did not complete the first questionnaire, and the results for round one were calculated based on the input of 24/26 panellists. Considering the high level of consensus reached after the second questionnaire round, with only one restaging item not achieving consensus, this item was addressed during manuscript revision with feedback from all panellists.

### Future perspectives

The field of rectal cancer staging and treatment is a dynamic field with significant changes expected in the coming years. One major development is the use of alternative neoadjuvant strategies. For early-stage tumours (up to 5 cm and cN0-1 < 8 mm), the OPERA trial demonstrated that combining chemoradiation with either an external radiotherapy boost or contact brachytherapy can drastically increase complete response rates up to 81% at 3 years [40]. At the other end of the spectrum total neoadjuvant therapy (TNT; i.e., combining (chemo)radiation with 4–6 cycles of chemotherapy) has emerged as an alternative for chemoradiation alone in “very” high risk locally advanced rectal cancer, including (combinations of) cT4b, EMVI+, (bilateral) lateral node involvement, tumour deposits, and MRF+ disease [41, 42]. TNT has been associated with higher rates of organ preservation and a reduced risk of distant metastases, albeit without improving overall survival [41–43]. Another exciting development is immunotherapy. Particularly in mismatch repair-deficient tumours, response rates have been exceptionally high. Immunotherapy is likely to become an integral part of rectal cancer management over the next 5–10 years [44–46]. These evolving strategies will likely lead to a more individualised approach to neoadjuvant strategies, where radiologists will play a crucial role in patient selection. As organ preservation becomes a key goal of treatment, complete response will become more prevalent. Therefore, the radiologist’s focus in restaging will have more emphasis on identifying potential complete responders.

The introduction of new neoadjuvant strategies also raises important questions about the criteria for response evaluation: are current assessment criteria, initially developed for conventional chemoradiation, still applicable in these new therapeutic contexts? Preliminary clinical experience shows that response may vary between neoadjuvant strategies, necessitating the development of new criteria [47].

Initial evidence suggests that both baseline imaging and serial MRI evaluations during neoadjuvant treatment may help to identify patients who are likely to respond either very well or very poorly [48, 49]. Such insights could inform adaptive treatment strategies, for example, by intensifying therapy (e.g., radiotherapy boost) in good responders or modifying the treatment strategy in those showing limited response. Criteria for such clinical decisions, based on baseline MRI and evolving response at MRI, are currently lacking but are expected to become available in the future.

In the foreseeable future, integrated diagnostics will likely play a central role in the clinical management of rectal cancer. Imaging alone will no longer suffice for clinical decision-making. To accurately select the right treatment for the right patient, detect local recurrence and distant metastases early, and monitor treatment response, clinical data, laboratory results (including circulating tumour DNA), histopathology and genomic profiling should be integrated with imaging. This paradigm shift will be practice-changing not only for patients and their treating physicians but also for radiologists. As radiologists, we must stay up to date with these innovations and doing so will require radiologists to expand their knowledge beyond the realm of imaging.

## Supplementary information

Supplementary Information

## Abbreviations

(c)CR: (Clinical) complete response
ADC: Apparent diffusion coefficient
CRT: Chemoradiation/chemoradiotherapy
DWI: Diffusion-weighted imaging
EMVI: Extramural venous invasion/extramural vascular invasion
ESGAR: European Society of Gastrointestinal and Abdominal Radiology
LLND: Lateral lymph node dissection
MDT: Multidisciplinary team
MRF: Mesorectal fascia
mrTRG: MRI tumour regression grade
RAM: RAND-UCLA appropriateness method
TD: Tumour deposit
TNT: Total neoadjuvant therapy
W&W: Watch-and-wait
yc-suffix: represents restaging of a feature after neoadjuvant treatment based on clinical and/or imaging assessment—e.g., ycT, ycEMVI
